# Non-Hodgkin's Anaplastic Large T-Cell Lymphoma: A Case Report

**DOI:** 10.7759/cureus.63040

**Published:** 2024-06-24

**Authors:** Gabriele A Halpern, Luiza Miziara Brochi, Cintia Gomes, Gabriela Lachter Zusman, Fabiane Carvalho de Macedo, Juliana Annete Damasceno Rodrigues

**Affiliations:** 1 General Practice, University Center of Volta Redonda, Volta Redonda, BRA; 2 General Practice, Universidade de Uberaba, Uberaba, BRA; 3 General Practice, Universidade Federal de Santa Maria, Santa Maria, BRA; 4 General Practice, Fundação Técnico Educacional Souza Marques, Rio de Janeiro, BRA; 5 Pathology, Fonte Medicina Diagnóstica, Niterói, BRA; 6 Hematology and Oncology, Centro Universitário de Volta Redonda - UniFOA, Volta Redonda, BRA

**Keywords:** lymphoma, case report, anaplastic large-cell lymphoma, large cell lymphoma, non hodgkin's lymphoma

## Abstract

Anaplastic large T/null cell lymphoma (ALCL) is an aggressive non-Hodgkin’s lymphoma (NHL) that most commonly affects young men. Herein, we present a case of a 32-year-old male patient in severe condition with ulcerated right axillary adenopathy, diffuse subcutaneous nodules, and sepsis. He was admitted to the ED, where a bone marrow aspirate and biopsy confirmed the diagnosis of ALCL. The immunohistochemical examination demonstrated neoplastic cells with immunopositivity with antibodies CD3 (focal), CD30 (diffuse), protein ALK-1 (diffuse), and epithelial membrane antigen (EMA) (multifocal). Appropriate chemotherapy treatment was done, and the patient showed a complete response. This article aims to report a rare subtype of NHL to increase awareness and bring up a discussion about the clinical presentation and diagnostic features of ALCL. Moreover, we discuss treatment regimens that are currently used and have shown reasonable disease remission rates.

## Introduction

Lymphomas are a group of neoplasms originating from the lymphoid tissue of the tonsils, thymus, spleen, and lymph nodes [1]. Regarding classification, lymphomas can be divided into Hodgkin's lymphomas (LH) and non-Hodgkin's lymphomas (NHL). The diagnosis and subtyping require clinical, serological, morphological, and potentially cytogenetic information. Lymph node biopsy is also needed for definitive subtyping in most cases. The subtypes of NHL depend on tumor growth (aggressive or indolent) and the type of lymphocytes involved (B-cells, T-cells, or natural killer cells (NK) [1].

Anaplastic large T-cell NHL is relatively rare, and patients usually have lymphadenopathy or an extranodal mass and may have fever, night sweats, and weight loss. Because of several factors, the patient may present an atypical and more severe form of the disease, leading to a particular difficulty for the doctor regarding the diagnosis. It is essential that, at this moment, the doctor can consider the most diverse differential diagnoses and perform the correct management of the patient to provide the best possible care.

## Case presentation

**Figure 1:**
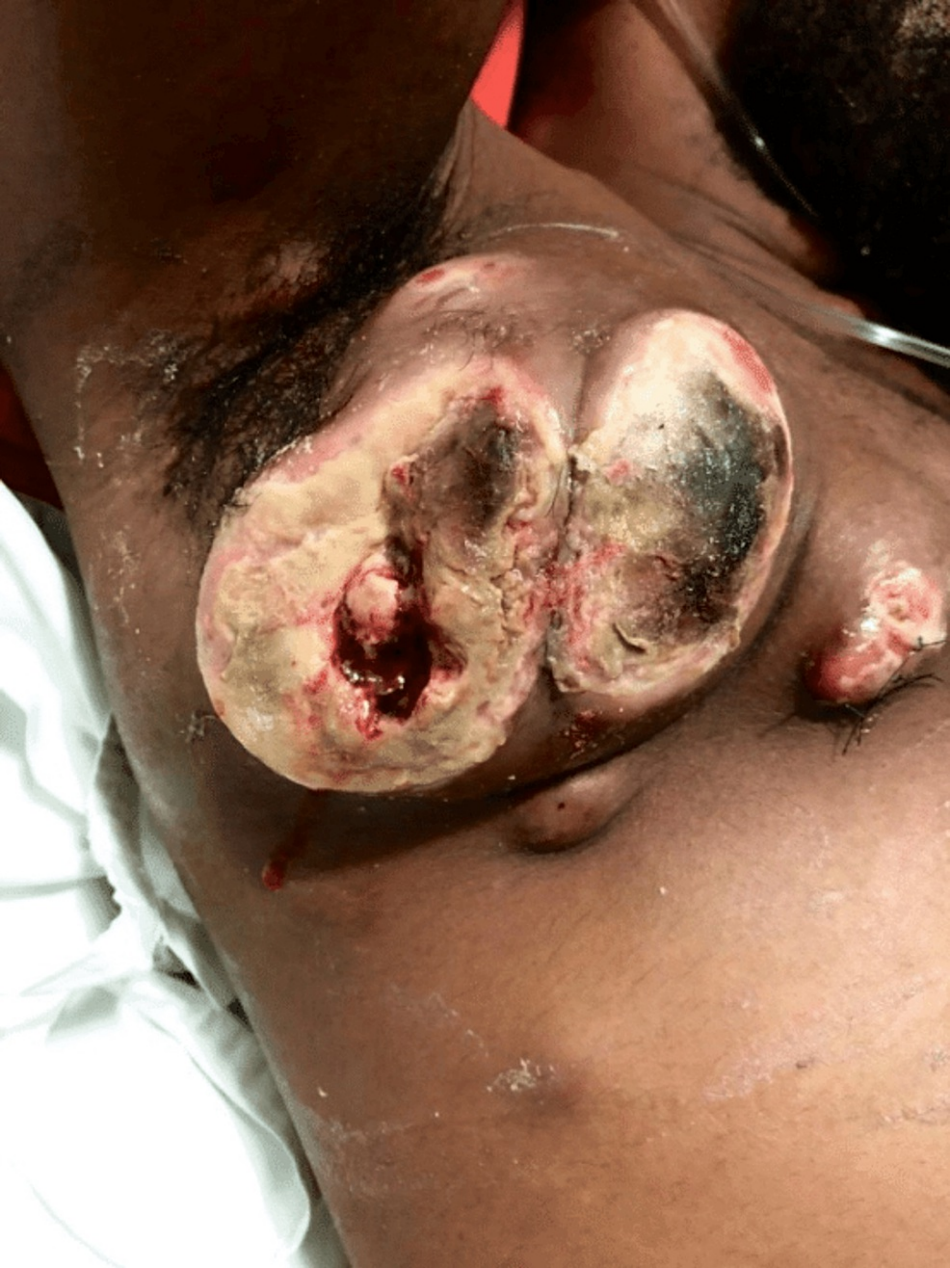
Right axillary mass The patient's right axilla with an ulcerated mass and necrotic areas at hospital admission.

A 32-year-old Brazilian man was admitted to the ED in a serious, tachypneic, and tachycardic state. He was previously healthy and had no significant family history or previous interventions. A physical examination showed an ulcerated right axillary mass with necrotic areas and signs of secondary infection (Figure 1). Hepatomegaly and diffuse subcutaneous nodules were also present throughout the body, causing pain and exhibiting an erythematous surface.

The patient worked on a ship; therefore, he could not see a doctor when symptoms first started. Initially, he believed that the wound was furunculosis secondary to deodorant use. When the lesion ulcerated, he asked the superiors of the crew to return to the continent and sought a hospital unit reporting a progressively growing mass in the last four months. The patient's right axilla had an ulcerated mass and necrotic areas at hospital admission, and he also reported having had all B symptoms (fever, weight loss, night sweats), but he could not specify when these symptoms started and how much weight he had lost. The physician who performed the first care suspected neoplasia and requested a hospital transference.

Because of the severity of the case, he was then admitted to the ICU. On the first day of ICU admission, he was pale, hypotensive, tachycardic, and septic. On the same day, he started antibiotic therapy with linezolid and meropenem and other supportive treatments, such as fluid resuscitation and RBC transfusion.

At the time of the diagnostic investigation, some pathologies were part of the differential diagnosis, such as Bacillary Angiomatosis, but it was unlikely because of the absence of abdominal pain, nausea, vomiting, diarrhea, and bone involvement. Another differential diagnosis was Yersinia enterocolitica, which became unlikely because the patient did not have vomiting, diarrhea, or joint involvement.

In addition, histoplasmosis, lymph node tuberculosis, blastomycosis, sporotrichosis, metastatic carcinoma, metastatic melanoma, Hodgkin’s lymphoma, and other diseases were also considered. Some of them, histoplasmosis, blastomycosis, and sporotrichosis seemed to be unlikely because of epidemiological history and clinical signs and symptoms since the place where the patient used to live was not endemic and he did not have contact with wild animals or cats. lymph node tuberculosis, metastatic carcinoma, melanoma, and Hodgkin’s lymphoma were only excluded after subsequent diagnostic examinations (biopsy and bone marrow aspiration).

Because of the wound severity and size, the medical team decided to perform a frozen biopsy of the right axillary arm when a histopathology corresponding to a lymphoma was found.

**Figure 2:**
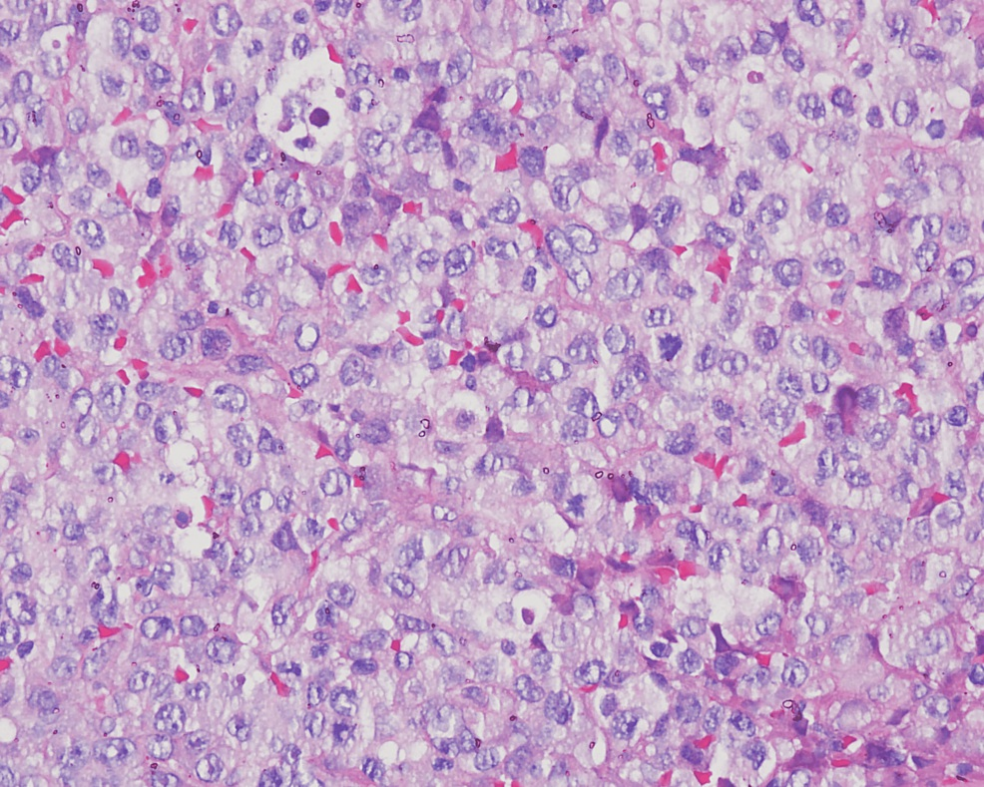
Biopsy of the patient's right axillary mass A biopsy of the patient's right axillary mass showing diffuse proliferation of large cells with evident, irregular, and eosinophilic nuclei (hematoxylin and eosin stain (HE), 400X).

**Figure 3:**
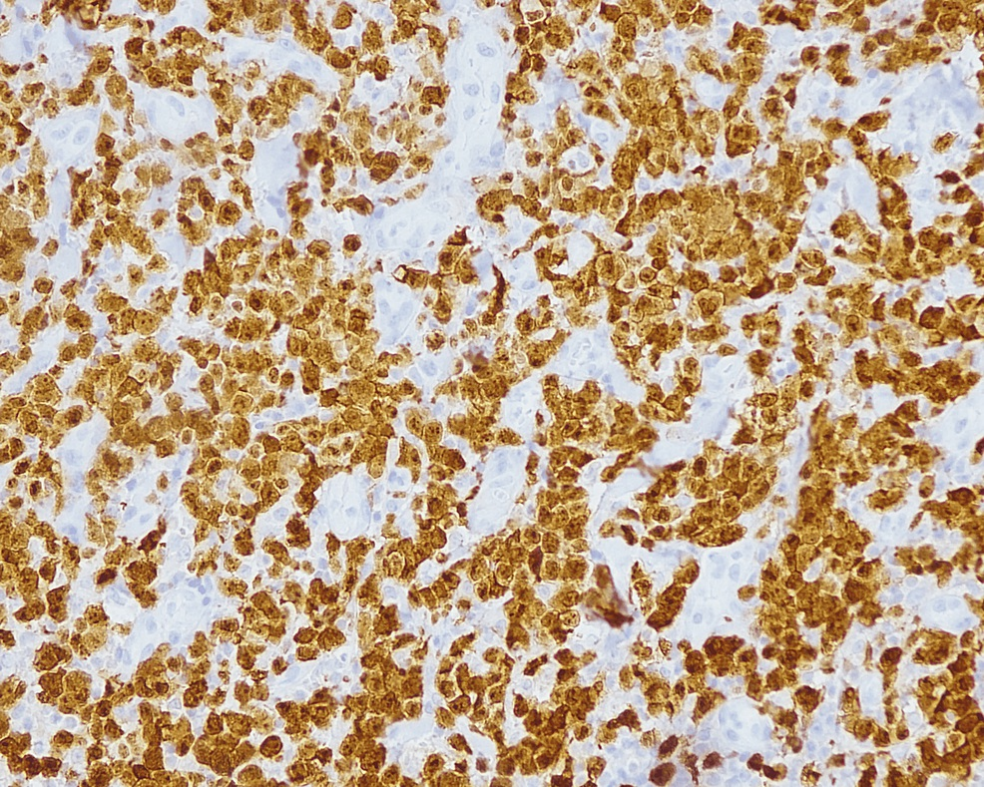
Immunohistochemical examination of the patient's right axillary mass Immunohistochemical stain: the neoplastic cells are positive for ALK1 cytoplasmatic positivity with membrane accentuation (ALK1, 200X).

After stabilization of the patient, a biopsy of the patient's right axillary mass was performed (Figure 2). Immunohistochemical examination (Figure 3) demonstrated neoplastic cells with the following immunostaining profile: immunopositivity with antibodies CD3 (focal), CD30 (diffuse), protein ALK-1 (diffuse), and epithelial membrane antigen (EMA) (multifocal); immune negativity with antibodies to CD20, CD10, and cytokeratins cocktail.

According to microscopy, it was concluded that it was a non-Hodgkin’s lymphoma of large anaplastic cells. 

However, because of the severity of the condition, even before the definitive histopathological result, chemotherapy treatment was started with the following medications: cyclophosphamide (CHP) 750 mg/m² IV on the first day (D1), doxorubicin, 50 mg/m² IV, on D1, vincristine, 1.4 mg /m² IV, on D1 (maximum dose of 2 mg) and prednisone, 100 mg orally, for five days (D1 to D5). The day after the chemotherapy infusion, there was a substantial reduction in the size of the axillary mass and a significant clinical improvement in the condition.

After completion of immunohistochemistry, the chemotherapy regimen was changed to brentuximab + CHP: brentuximab 1.8 mg/kg on D1, CHP 750 mg/m2 on D1, doxorubicin 50 mg/m2 on D1, and prednisone 100 mg orally from D1 to D5, every 21 days. At the end of treatment, there was a complete response, and the patient was kept under follow-up. The patient did not experience any side effects or intolerability signs during treatment. As he was undergoing chemotherapy at the hospital, the treatment adherence was excellent.

At the end of the first cycle, the subcutaneous nodules had shown impressive improvement, and by the end of the third cycle, all of them had disappeared. After six cycles of chemotherapy, the patient achieved a complete response, with the lesions and extranodal signs disappearing. Additionally, hepatomegaly showed total improvement by the end of the treatment. Unfortunately, the hospital did not have access to PET CT to compare before and after treatment, and the patient was not submitted to other imaging tests before starting the treatment because of his unstable clinical condition caused by sepsis.

There were no relapses before the complete response. Following this, the patient was monitored at the hospital's hematology service for two years after treatment, and, during this period, there were no signs of recurrence.

## Discussion

NHL is the fourth most common neoplasm in the United States, excluding non-melanoma skin cancer. Moreover, it is the ninth leading cause of cancer-related death in males and seventh in females, contributing to 5% of cancer-related deaths. High-grade lymphomas account for approximately 50% of all NHL cases, including anaplastic large-cell lymphoma [2].

In the present article, we presented the case report of a 32-year-old Brazilian man with anaplastic large-cell NHL. At the time of the diagnosis, the patient had a large and ulcerated axillary mass in addition to poor general condition. Axillary lymph node biopsy was performed, where neoplastic cells with CD30 marker positivity were found.

Most patients with NHL present with non-painful peripheral lymphadenopathy and may have fever, night sweats, and weight loss. Adenopathy predominates in the cervical, supraclavicular, or inguinal chains, but any chain can be affected [3]. Therefore, biopsy should be recommended whenever a lymph node has the following characteristics: size greater than one centimeter, supraclavicular location, multiple nodal sites, progressive growth, hardened consistency, or adherence to deep planes [2].

Patients who present with subcutaneous nodules in the context of suspected or diagnosed lymphoma usually lead the clinician to suspect subcutaneous panniculitis-type T-cell lymphoma (SPTCL), which is an extranodal manifestation of the disease. SPTCL is a rare subtype of primary cutaneous T-cell lymphoma that mimics panniculitis, an inflammation of the adipose tissue that can affect patients with inflammatory or autoimmune diseases, for example [4].

Among T/NK cell lymphomas, there is anaplastic large T-cell lymphoma, as presented by the patient reported in the case. Such a type of lymphoma presents, as a diagnostic criterion, the presence of the CD30 antigen in patients with a genetic translocation mutation, most common between chromosomes 2 and 5 [4], which often leads to hyperexpression of anaplastic lymphoma protein kinase (ALK) and constitutes about 2%-3% of NHLs [5].

The most significant differences between ALCL ALK-positive and ALCL ALK-negative are clinical features and prognostic. The ALK-positive cases mainly affect younger patients and are more likely to present in extranodal sites compared to ALK-negative cases. However, prognosis and survival rates are significantly better in ALK-positive ALCL compared to ALK-negative cases [6].

According to the American Cancer Society, non-Hodgkin’s lymphoma (NHL) can manifest at any stage of life, with a notable prevalence among children, teenagers, and young adults. However, the likelihood of NHL onset rises with age, and over 50% of individuals receive their initial diagnosis at the age of 65 or beyond. Systemic symptoms and abnormal laboratory conditions are often found. The most found laboratory alterations are lactate dehydrogenase (LDH) elevation (37%), anemia (27%), and thrombocytopenia (10%) [7]. Regarding the symptoms, the most commonly found are B symptoms, which consist of fever, night sweats, and weight loss, as well as the involvement of lymph nodes and extranodal sites [8].

The difference in event-free survival (EFS) for younger patients and ALK-positive ALCL treated with cyclophosphamide, hydroxydaunomycin, oncovin, and prednisone (CHOP) or cyclophosphamide, doxorubicin, etoposide, vincristine and prednisone (CHOEP) within trials is dramatic (three-year EFS for CHOEP patients was 91.2% vs. 57.1% for patients treated with CHOP, P=0.012). The results above show a relevant prognostic difference within the addition of etoposide to CHOP, improving response rates, mostly in younger patients. This represents a strong and relevant reason why more patients with anaplastic large-cell lymphoma may be able to undergo an autologous or allogeneic transplant after CHOEP. Several institutions actively pursue these two transplant modalities to improve the disease's prognosis [9].

Brentuximab chemotherapy is another option for treatment. It induces various responses, largely positive in nature and variable between NHL subtypes [10]. With additional and properly powered prospective studies, it may prove to be a strong candidate for treating various CD30+ malignancies [11]. Some phase 3 trials have already proven the efficacy of brentuximab in ALCL [12].

The CHOP regimen has long been considered the standard in peripheral T-cell lymphomas and can still be used when brentuximab is unavailable. The addition of etoposide, 100 mg/m² IV, from D1 to D3 to CHOP, may be considered in individuals younger than 60 years of age and normal LDH because it is associated with better outcomes [13]. The ECHELON-2 study has significantly advanced treatment approaches for systemic anaplastic large cell lymphoma (sALCL) and other CD30-expressing peripheral T-cell lymphomas (PTCL). The study revealed that frontline treatment with brentuximab vedotin (BV) alongside cyclophosphamide, doxorubicin, and prednisone (A+CHP) surpasses the standard CHOP regimen.

With a median 36.2-month follow-up for progression-free survival (PFS), A+CHP demonstrated a significant advantage over CHOP, as indicated by a hazard ratio (HR) of 0.71 (95% CI: 0.54, 0.93), and P=0.01. The median PFS was notably prolonged to 48.2 months (95% CI: 35.2, not evaluable) for A+CHP compared to 20.8 months (95% CI: 12.7, 47.6) for CHOP.

Regarding overall survival (OS) with a median 42.1-month follow-up, the HR of 0.66 (95% CI: 0.46, 0.95), and P=0.02 underscored the superior efficacy of A+CHP. Notably, the median OS was not reached for either treatment arm, indicating a sustained benefit.

A+CHP marks a significant milestone as the first regimen demonstrating an OS benefit over CHOP in this patient population. These results are crucial for clinicians and researchers, forming a robust foundation to advance treatment strategies and enhance outcomes for individuals with sALCL and CD30-expressing PTCL. This report provides updated results with a median follow-up of 44.3 months for PFS and 55.5 months for OS, further affirming the long-term efficacy and superiority of the A+CHP regimen [14].

In this case, the patient's treatment was delayed because of his job on a board, which undoubtedly contributed to the severity of the disease, leading to ICU admission because of septic shock and hemodynamic instability. However, even with these complications, he followed up with an excellent treatment response and no disease recurrence.

## Conclusions

NHL poses a significant threat, particularly to young men, with a heightened risk of morbidity and mortality. Early detection is crucial, as it often manifests initially through B symptoms such as fever, significant weight loss over six months, and night sweats. Given the substantial risk of mortality, a prompt lymph node biopsy is imperative for confirming the diagnosis and determining the disease's stage, facilitating the timely initiation of appropriate treatment. Currently, the CHOEP regimen is widely employed as the primary treatment. Recent studies suggest brentuximab as a promising frontline treatment for ALCL and other CD30+ malignancies. Despite these advancements, discovering a preventive measure for lymphoma onset remains an ongoing challenge. Recognizing symptoms and ensuring a swift and accurate diagnosis is paramount for implementing effective treatment strategies and improving patient outcomes.
